# Rimplasty: An Arthroscopic Technique for Patients With Isolated Patellar Chondromalacia Based on Autologous Matrix‐induced Chondrogenesis+ and Limited Trochleoplasty

**DOI:** 10.1002/atn2.70100

**Published:** 2026-05-26

**Authors:** Tomasz Piontek, Bartosz Bąbik, Jakub Bąbik, Artur Banach

**Affiliations:** ^1^ Rehasport Clinic Poznan Poland; ^2^ Department of Spine Disorders and Pediatric Orthopedics, University of Medical Sciences Poznan Poland; ^3^ Department of Orthopaedics, Pediatric Orthopaedics and Traumatology Medical Centre of Postgraduate Education Warsaw‐Otwock Poland; ^4^ Doctoral School of Translational Medicine, Centre of Postgraduate Medical Education Warsaw Poland; ^5^ Faculty of Architecture Warsaw University of Technology Otrębusy Poland; ^6^ MicroBioRobotic Systems Laboratory Institute of Mechanical Engineering École Polytechnique Fédérale de Lausanne Lausanne Switzerland

## Abstract

Anterior knee pain is a common problem that can be difficult to treat. One potential cause of these symptoms is supratrochlear rim, which has been linked to isolated patellar chondromalacia in patients with patella alta and without any history of patellar dislocation or subluxation. In cases where the rim does not exceed 3 mm, the rim resection technique was previously described. This technical note outlines rimplasty, an arthroscopic technique addressing the biomechanical etiology of patellar chondromalacia by modifying patellofemoral joint kinematics and reconstructing patellar cartilage. The described technique specifically targets cases in which the supratrochlear rim exceeds 3 mm in height on imaging.

## Graphical abstract

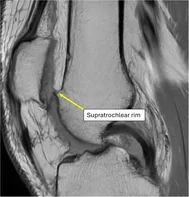

**VIDEO 1:** Surgical technique of rimplasty. This is a video of arthroscopic rimplasty. In this video, we show a rimplasty procedure in a young adult male with persistent left knee pain in the anterior compartment that did not improve with prolonged rehabilitation. Before surgery, he complained of pain during squatting and while walking up the stairs and of pain that limited his daily activities. Before surgery, it is crucial to confirm the supratrochlear rim on the T1‐weighted magnetic resonance imaging slices in the sagittal view. Supratrochlear rim is delineated, and its height is measured on the sagittal slice. A line is drawn parallel and in line with the frontal femoral cortex; then, a perpendicular line is drawn starting at the top of the peak of the supratrochlear rim. The height of the supratrochlear rim is the distance between the peak of the supratrochlear rim and the line parallel to the frontal femoral cortex. The rim height should exceed 3 mm. In this case, a patellar chondral defect was also found on the sagittal magnetic resonance imaging slice near the supratrochlear rim. The patient was then deemed eligible for a rimplasty procedure. The procedure is shown on the patient's left limb. The patient is lying supine with a tourniquet in place, and the surgical field and drapes are prepared in a sterile manner. The surgeon is seated, with the patient's foot resting on their lap. The patient's knee is flexed to 90°. The surgeon is facing the patient's head (Position I). The anterolateral portal is created, followed by opening the anteromedial portal under direct visualization. Then, 2 additional portals are placed—the first suprapatellar portal on the lateral side and the second suprapatellar portal on the medial side. The suprapatellar medial portal should be wider to allow a 5 mm osteotome to be easily introduced into the joint. To choose an appropriate site, the suprapatellar medial portal needle should be introduced to check if the angle at which the osteotome will be introduced during rimplasty is correct. First, diagnostic arthroscopy is performed with an examination of all knee compartments. Concomitant injuries and pathologies are addressed first. Then, with the knee fully extended and a surgeon standing beside the patient's thigh facing the patient's feet (Position II), the supratrochlear rim is addressed. The supratrochlear lateral portal is used for viewing, and the supratrochlear medial portal is used as a working portal. Approximately 10 mm of the anterior femoral cortex is visualized with an arthroscopic shaver and coagulation to serve as a reference for the extent of rimplasty. A 5 mm osteotome is introduced, and the chondral flap covering the supratrochlear rim is carefully elevated. Then, rimplasty is performed using the anterior femoral cortex as a reference line for the extent of resection. Care must be taken not to breach or cut off the previously elevated chondral flap. An arthroscopic burr (5.5 mm DYONICS ELITE Abrader, Smith & Nephew, Watford, UK) is used to smooth the rimplasty site. To facilitate healing, care is taken to preserve approximately 1 mm of subchondral bone beneath the cartilage. The chondral flap is carefully mobilized and pressed against the frontal femoral cortex at the resected site using an arthroscopic hook or a blunt instrument. In “Position I,” the first Healicoil Knotless PK suture anchor (Smith & Nephew, Watford, UK) is placed through the anteromedial portal, with the anterolateral portal used for visualization. The anchor is aligned with the trochlear groove and beneath the cartilage surface to avoid joint surface conflict. Two ETHICON Coated VICRYL 6 (Johnson & Johnson International, New Brunswick, NJ) sutures are used for this anchor (4 strands are needed for further stabilization: 2 strands for the second and 2 for the third anchor). The surgeon returns to “Position II” to place the second Healicoil Knotless PK suture anchor (Smith & Nephew, Watford, UK) 10 mm proximal and aligned with the trochlear groove. The third anchor is placed lateral to the second anchor, superior to the trochlear groove. For each anchor, 2 strands of ETHICON Coated VICRYL 6 (Johnson & Johnson International, New Brunswick, NJ) are used and tightened to stabilize the chondral flap after rimplasty. Patellar cartilage lesion is investigated and treated next. The extent of cartilage damage is determined with an arthroscopic hook. Preparation of the lesion is performed with front and sideward chondrectomes as needed (Chondrectom Extended Set, Biovico, Gdynia, Poland) to the margins of healthy cartilage, and uneven edges are smoothed with an arthroscopic shaver. The resection proceeds to the subchondral bone. For this step, the anterolateral portal is usually used as a viewing portal and the anteromedial portal as a working portal; however, depending on the location of the defect, the remaining portals can also be used. In dry arthroscopy, the arthroscopic autologous matrix‐induced chondrogenesis technique is used to treat the previously prepared lesion. Round (5 × 5 mm) circles cut from collagen membrane (Chondro‐Gide, Geistlich, Switzerland) soaked in bone marrow aspirate are introduced to the previously prepared chondral lesion on the patellar articular surface. Fibrin glue (Tissucol, Baxter, Warsaw, Poland) is used to stabilize the collagen membrane. No adhesions of synovium or soft tissue with glue are allowed. After this step, joint congruity is assessed through passive flexion and extension and visualized arthroscopically via the anterolateral or lateral suprapatellar portal. After confirming satisfactory fixation and alignment, the incisions are closed without drainage, and a layered dressing is applied. The procedure is complete. Video content can be viewed at https://doi.org/10.1002/atn2.70100.

Knee pain occurs in nearly 30% of young individuals presenting to medical care, and among them, the prevalence of anterior knee pain (AKP) reaches up to 50%.^1^, ^2^, ^3^ If not adequately treated, AKP may lead to progressive cartilage damage of the patella and eventually to degenerative changes of the patellofemoral joint (PFJ), accompanied by intensifying pain symptoms.^4^, ^5^

Potential contributing factors for patellar cartilage (PC) lesions include biomechanical dysfunction of the PFJ, patella alta, trochlear dysplasia, and morphological abnormalities of the femur's supratrochlear spur.^6^, ^7^, ^8^, ^9^, ^10^, ^11^, ^12^, ^13^ Outerbridge was among the first to highlight the association between PFJ pain and morphological irregularities of the proximal entrance to the trochlear groove, known as the supratrochlear rim in patients without trochlear dysplasia and history of patellar instabilities.^14^ Subsequent studies revealed that open surgical resection and contouring of this rim could significantly alleviate AKP in appropriately selected patients.^15^, ^16^ Banach et al. showed correlation between PC defects and the supratrochlear rim based on magnetic resonance imaging measurements.^17^ Recently, a method for removing supratrochlear rim and covering the defect using autologous matrix‐induced chondrogenesis has been proposed for rims with a height lower than 3 mm.^18^ For rims higher than 3 mm, in this technical note, we describe an arthroscopic procedure called rimplasty to remove the supratrochlear rim while sparing the overlying femoral cartilage.

## SURGICAL TECHNIQUE

### Indications for Rimplasty

**FIGURE 1:**
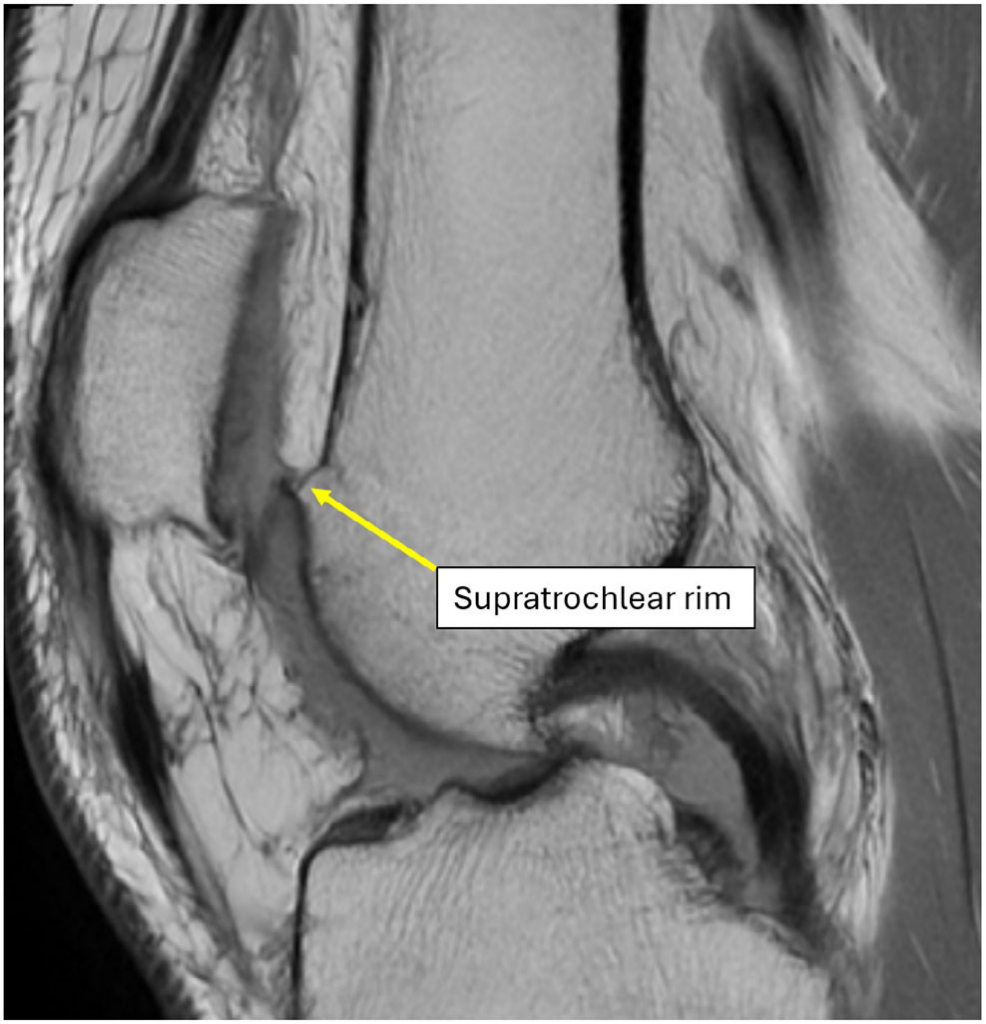
A sagittal MRI slice of the left knee joint in a patient with anterior knee pain, showing the medial supratrochlear rim. The rim is indicated by a yellow arrow. The depicted cross‐section is the plane where the anterior outline of the medial femoral condyle first becomes visible, along with the supratrochlear rim. MRI of the knee is necessary to confirm the presence of the rim before planning surgery and to assess any patellar articular cartilage damage. (MRI, magnetic resonance imaging.)

**FIGURE 2:**
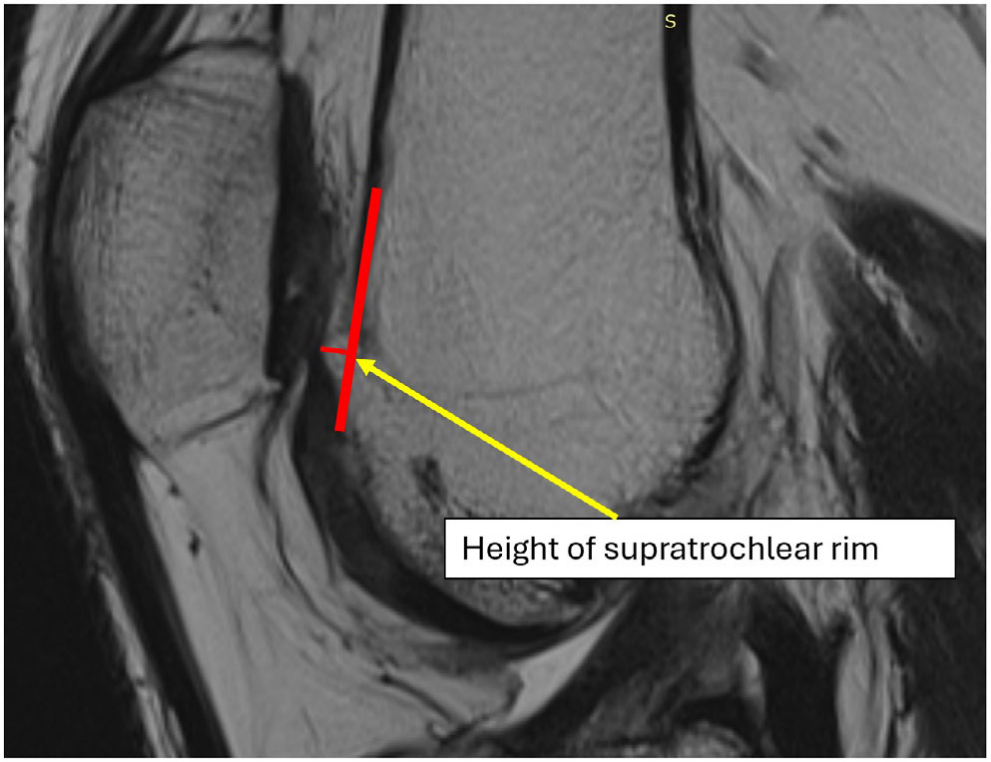
Image of the left MRI sagittal slice with a measurement of the supratrochlear rim height. The measurement is performed on a T1‐weighted sagittal MRI slice where the deepest point of the trochlear groove is visible. First, a reference line is drawn along the anterior femoral cortex. Then, from the highest point of the supratrochlear rim, a perpendicular line is extended to meet this reference line. The vertical distance between the rim's apex and the cortex line represents the rim height. These measurements are crucial for surgical planning. The patient will be considered for rimplasty if the height exceeds 3 mm. This MRI slice shows the right knee of a young male with anterior knee pain, pain during squatting, and a patellar chondral defect. Rehabilitation was unsuccessful, and the patient was subsequently prepared for surgery. (MRI, magnetic resonance imaging.)

The following are the indications for surgery: supratrochlear rim visible on magnetic resonance imaging in sagittal view (Figure 1), stable PFJ with no history of patellar dislocations, high‐riding patella (patellotrochlear index <0.125‐0.28),^19^, ^20^, ^21^ AKP during the first 30° of knee flexion, and supratrochlear rim height larger than 3 mm measured on sagittal view—see Figure 2 (if height is less than 3 mm, the patient should be considered for a different procedure^18^). Exclusion criteria include active joint infection.

### Surgical Technique

#### Rimplasty

**FIGURE 3:**
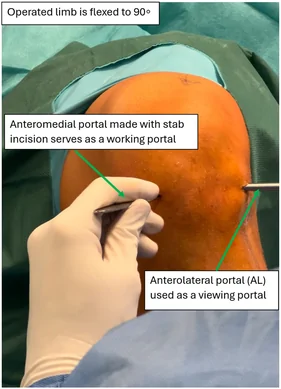
An image of the left knee joint, prepared for surgery, with sterile drapes in place. An AL portal is established and serves as a viewing portal. The AM portal is created with a stab incision under AL portal visualization. The knee is flexed to 90°, and the patient lies supine with a tourniquet applied. An arrow marks the location of the created AL and AM portal. The surgeon is seated, with the patient's foot resting on their lap. The patient's knee is flexed to 90°. First, diagnostic arthroscopy is performed, and all concomitant pathologies are identified and treated. The AL portal is then used as a viewing portal to guide the placement of the suprapatellar lateral and suprapatellar medial portals. (AL, anterolateral; AM, anteromedial.)

**FIGURE 4:**
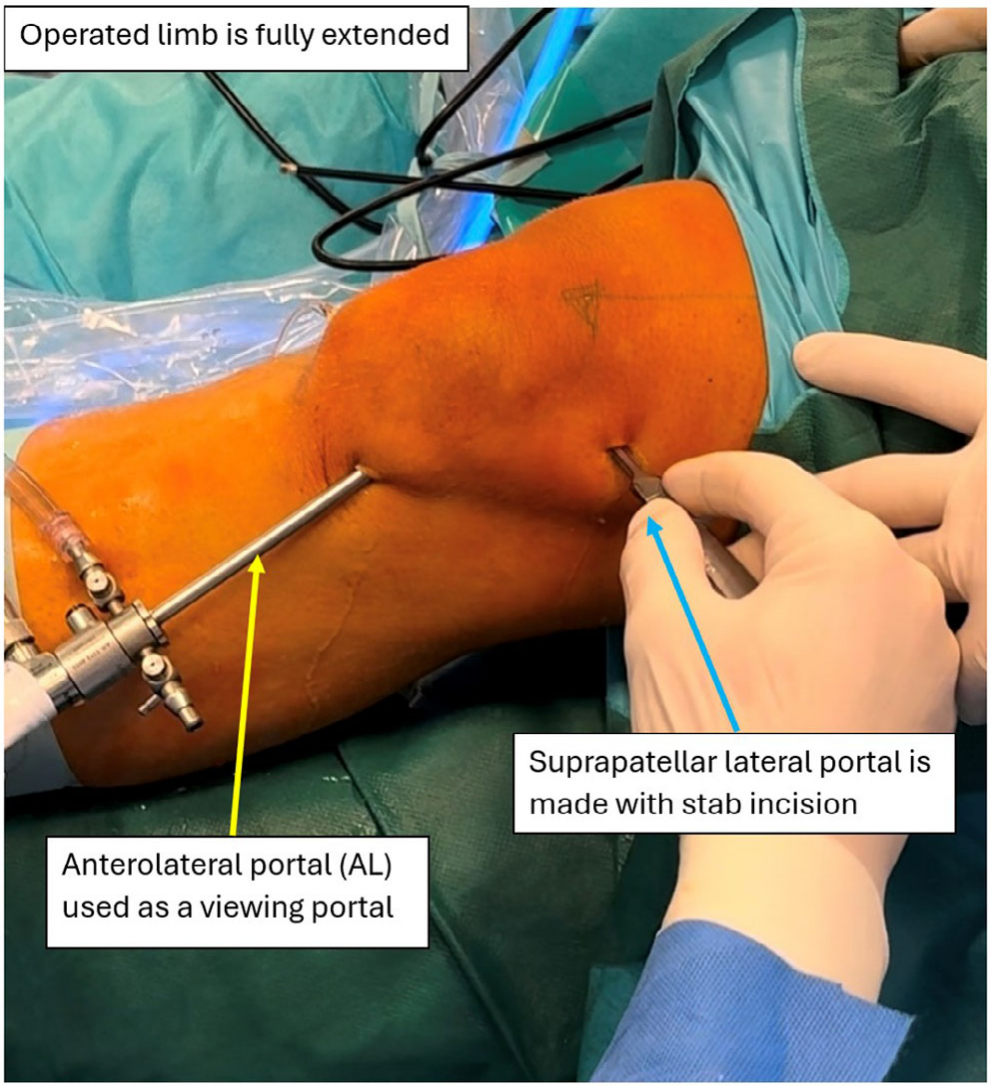
An image of the left knee joint prepared for surgery, with a sterile drape in place. It displays the creation of the suprapatellar lateral portal. The patient's knee is fully extended and resting on the operating table. The suprapatellar lateral portal is created with a stab incision, and the AL portal is used for visualization. Arrows indicate the AL portal and the suprapatellar lateral portal, which is created with a stab incision. This portal is further used as a viewing portal during preparation of the suprapatellar medial portal and during rimplasty. If preferred, it can also serve as a working portal. (AL, anterolateral.)

**FIGURE 5:**
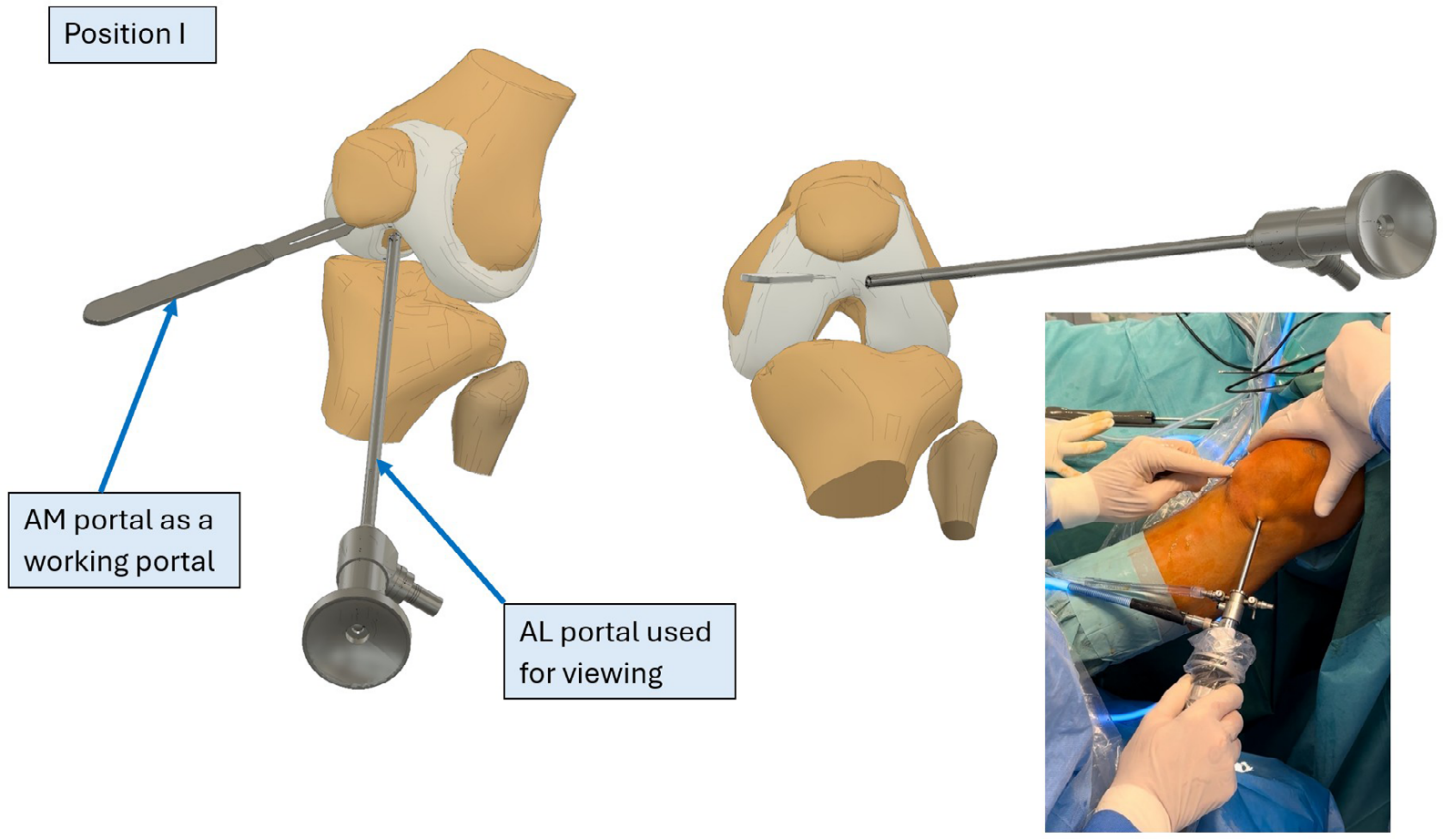
Technical illustration of “Position I,” in which the surgeon faces the patient's head, the AL portal provides visualization, and the AM portal serves as a working portal. This position is used for addressing concomitant injuries of the knee joint and inserting the first Healicoil Knotless PK suture anchor (Smith & Nephew, Watford, UK). Arrows indicate the arthroscope model introduced through the AL portal, which is used as a viewing portal, and a scalpel introduced through the AM portal, which serves as a working portal. In the lower‐right corner of the figure, a photograph of the left knee prepared for surgery with sterile drapes in place shows this position during the surgical procedure. The surgeon is seated, with the patient's foot resting on their lap. When creating the AM and AL portals and performing diagnostic arthroscopy of the medial, lateral, and anterior compartments of the knee, the knee is flexed to 90°. For visualization of the patellofemoral joint, the leg is fully extended and resting on the operating table. (AL, anterolateral; AM, anteromedial.)

**FIGURE 6:**
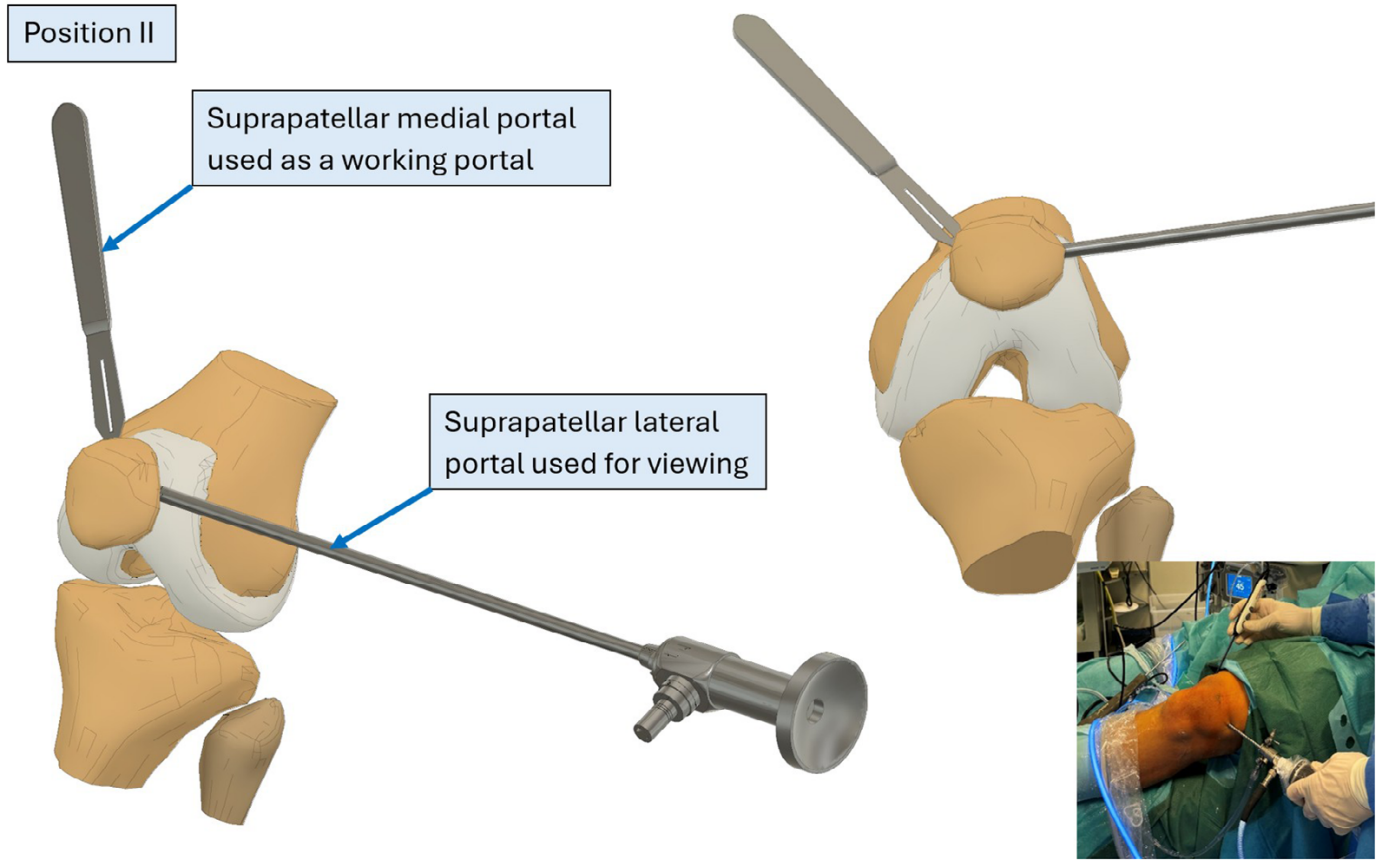
An image of “Position II,” in which the surgeon stands beside the operating table, and the patient's leg lies supine on it in full extension. The surgeon is facing the patient's feet. This position is used to address the supratrochlear rim and pathologies of the patellofemoral joint. Also, in this position, second and third Healicoil Knotless PK suture anchors (Smith & Nephew, Watford, UK) are inserted. Arrows mark the model of arthroscope introduced through the suprapatellar lateral portal, which is used as a viewing portal, and the scalpel introduced through the suprapatellar medial portal, which symbolizes a working portal. In the lower‐right corner of the figure, a photo shows the left limb with surgical drapes in full extension on the operating table, indicating this position during the surgical procedure. “Position II” facilitates access to the supratrochlear rim.

**FIGURE 7:**
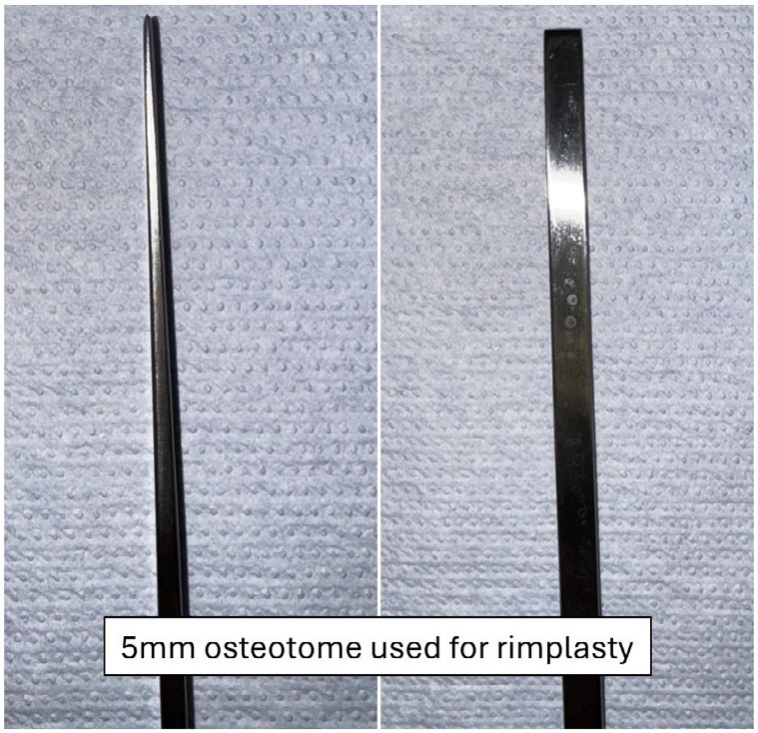
An image of a 5 mm osteotome, used for the rimplasty procedure. The right photo presents a coronal view, and the left photo presents a lateral view of the osteotome. The osteotome is introduced via the supratrochlear medial portal under visualization from the supratrochlear lateral portal, used to elevate the subchondral flap covering the supratrochlear rim, and then used for rimplasty. Care must be taken to prepare a wider supratrochlear medial portal to facilitate the introduction of the osteotome.

**FIGURE 8:**
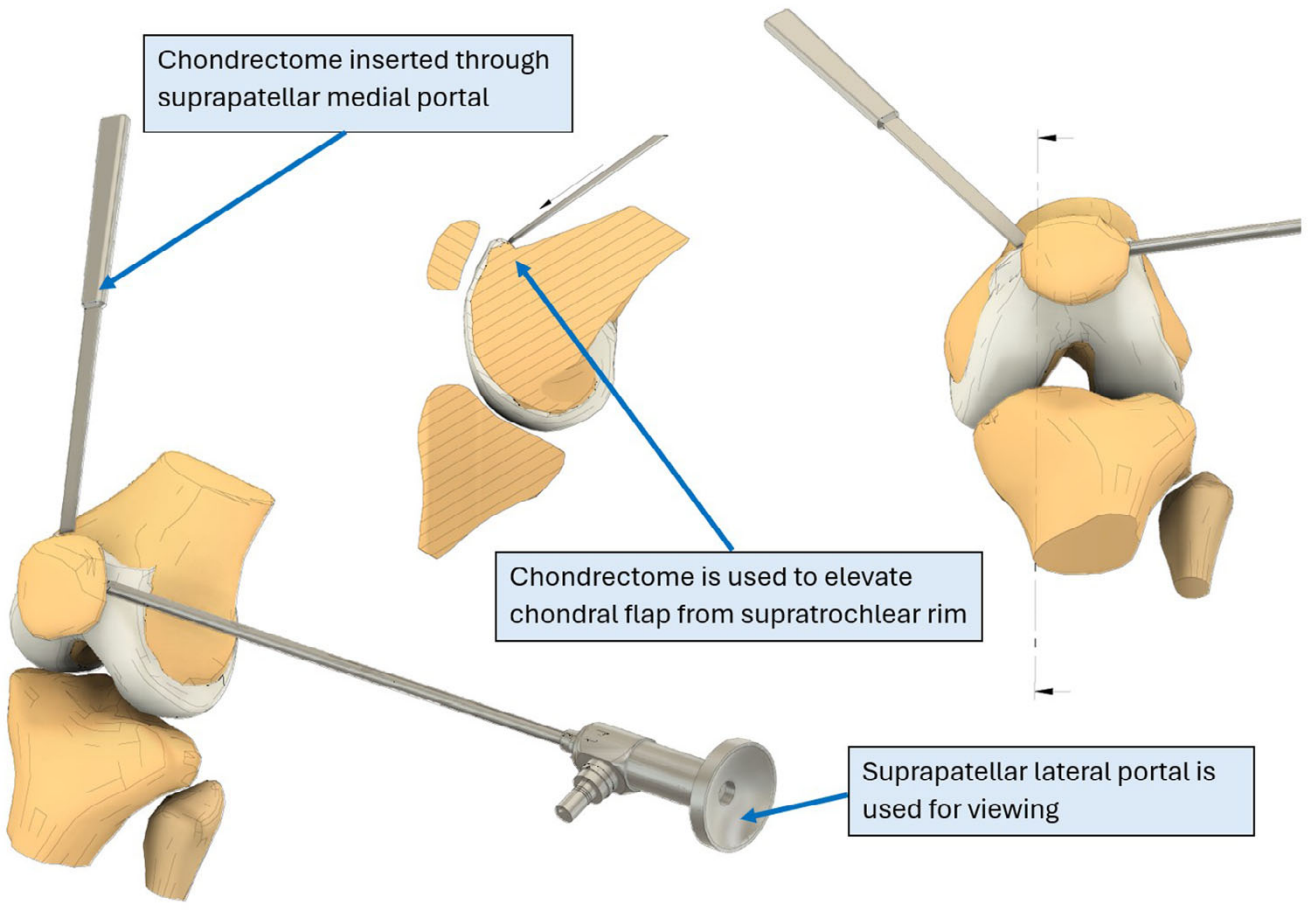
Technical drawing of the left knee joint illustrating chondral flap preparation. The chondral flap covering the supratrochlear rim is elevated with a 1 mm layer of subchondral bone exposing the supratrochlear rim. Care needs to be taken not to breach this chondral flap. Arrows indicate the arthroscope introduced through the suprapatellar lateral portal, which serves as a viewing portal, and the osteotome introduced through the suprapatellar medial portal. The central image represents a cross‐section of the image on the right, as indicated by the black line pointed to by black arrows. The chondral flap is elevated to expose the bony rim for further resection with the same osteotome and an arthroscopic burr (5.5 mm DYONICS ELITE Abrader; Smith & Nephew, Watford, UK). Finally, the previously elevated chondral flap is reinserted after bony rim excision. It is important that the chondral flap is flexible and mobile to make reinsertion possible.

**FIGURE 9:**
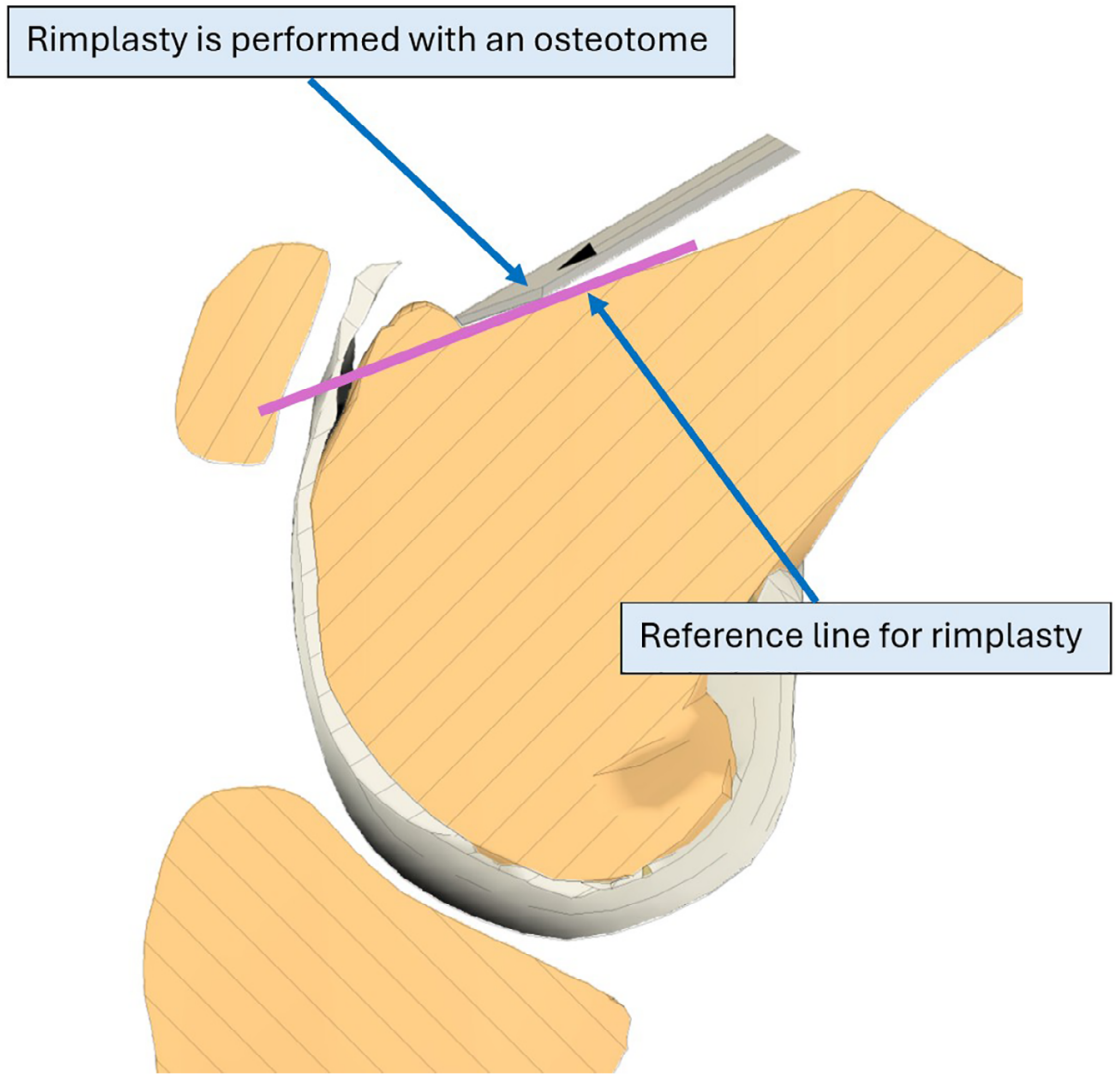
Technical drawing of the left knee undergoing rimplasty. After elevation of the chondral flap, a 5 mm osteotome is used for rim excision. After this step, rough edges are smoothed with an arthroscopic burr (5.5 mm DYONICS ELITE Abrader; Smith & Nephew, Watford, UK), and the site is prepared for reinsertion of the previously elevated chondral flap. Arrows mark the osteotome used for this procedure, and the outline of the frontal femoral cortex defines a reference line for the extent of rimplasty. Excision of the rim to the reference line is important to achieve a smooth transition between the frontal femoral cortex and the trochlea groove.

**FIGURE 10:**
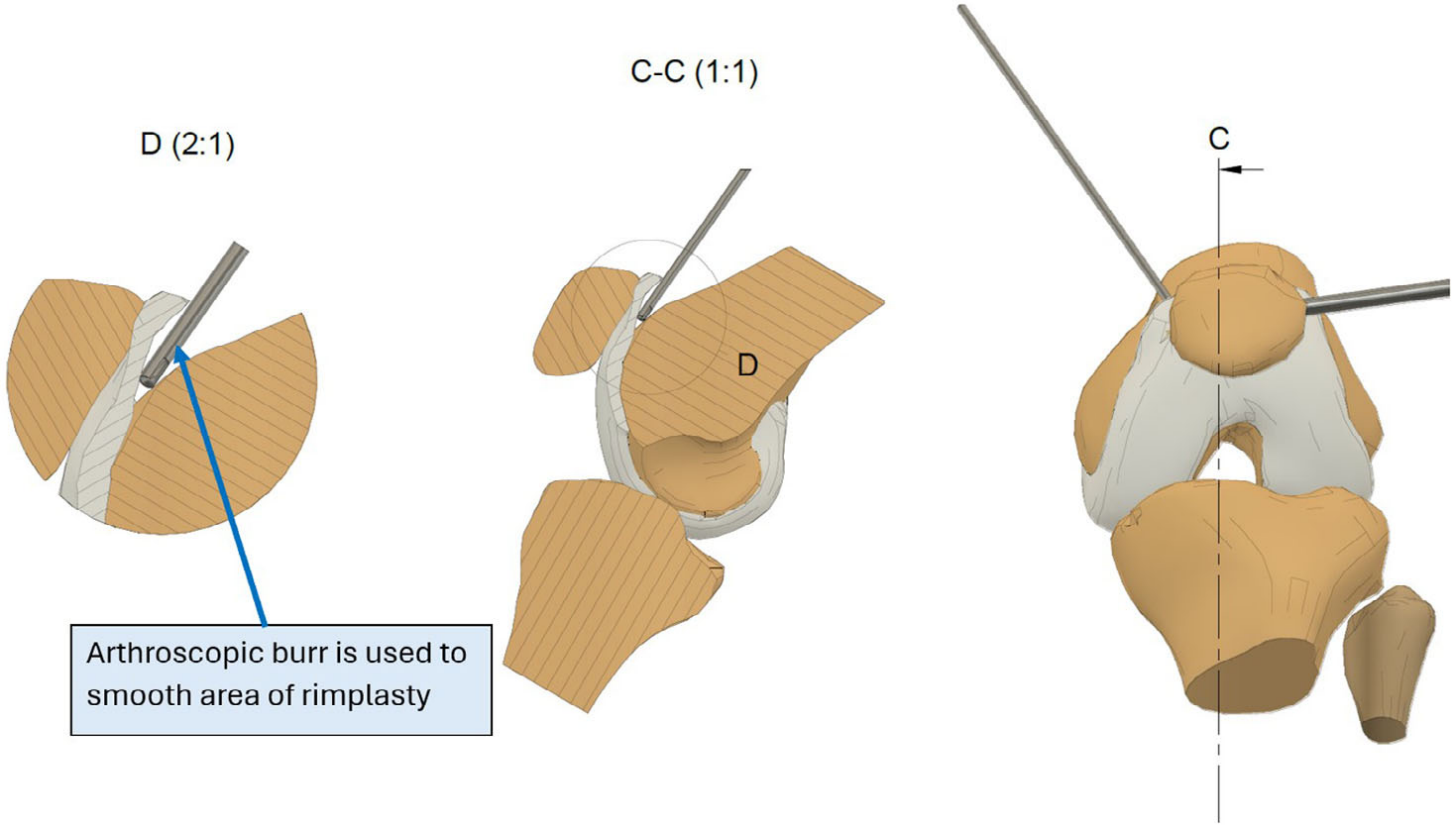
Technical drawing of the left knee in sagittal view (marked with C‐C). The rim area after excision with an osteotome is further exposed on the left side—technical drawing marked with D (2:1). The drawing on the right side of the figure shows the exact location of the aforementioned sagittal slice (line marked with C). The arrows indicate an arthroscopic burr, introduced through the suprapatellar medial portal, which is used to smooth the area of rimplasty and allow a smooth transition between the trochlear groove and the femoral cortex. This step follows chondral flap elevation and rimplasty with an osteotome. After this step, the previously elevated chondral flap is mobilized and then reinserted into the defect created by the bony rim excision.

**FIGURE 11:**
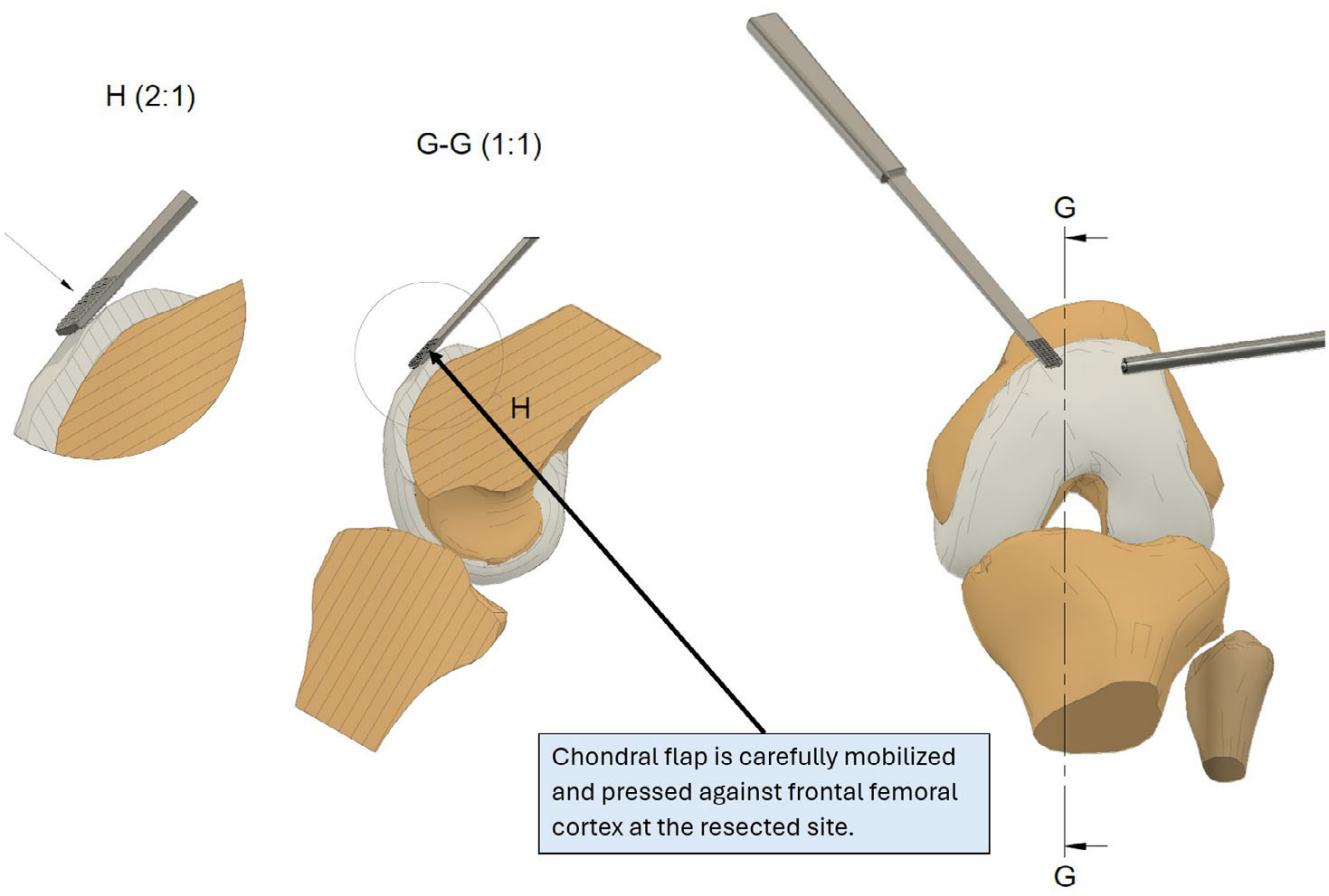
Technical drawing of the left knee in sagittal view (marked with G‐G). The area of rim after excision with an osteotome is further exposed on the right side—technical drawing marked with H (2:1). Drawing on the right side of the figure shows the exact location of the aforementioned sagittal slice (line marked with G). Arrows mark a blunt tool introduced through the suprapatellar medial portal, which is used to carefully mobilize and press previously elevated chondral flap against the frontal femoral cortex at the site of rimplasty. After rimplasty, the chondral flap will be reinserted into the area. Care must be taken not to break the chondral flap and to achieve a smooth transition between the frontal femoral cortex and the trochlear groove to allow the patellar slide with no conflict.

The surgical technique is presented in Video 1. The patient is under spinal anesthesia, lying supine, and a tourniquet is placed on the proximal thigh and inflated to 300 mmHg. A sterile drape is used, and the operative field is prepared using a standard aseptic technique. A standard anterolateral (AL) portal is created using a stab incision with a scalpel, followed by the creation of an anteromedial portal under direct arthroscopic visualization through the AL portal (Figure 3). First, all concomitant intra‐articular pathologies are treated. A spinal needle is introduced with the knee fully extended to identify the optimal location for the lateral suprapatellar portal under arthroscopic visualization via the AL portal. The lateral suprapatellar portal is then established with a stab incision (Figure 4). The surgeon changes position from standing or sitting beside the patient's shank, facing the patient's head (Position I) (Figure 5) to standing beside the patient's thigh, facing the patient's feet (Position II) (Figure 6). Next, a spinal needle is introduced to localize the medial suprapatellar portal under direct visualization through the suprapatellar lateral portal. This portal, created with a stab incision, is made slightly wider to allow insertion of the appropriate osteotome. The supratrochlear rim and anterior femoral cortex are visualized and cleared using an arthroscopic shaver and coagulation until the bony rim is free of synovium and fat pad. At least 10 mm of the anterior femoral cortex should be exposed. A 5 mm osteotome (Figure 7) is introduced, and the chondral flap covering the supratrochlear rim is carefully elevated (Figure 8). Then, rimplasty is performed using the anterior femoral cortex as a reference line of the extent of resection without X‐ray assistance (Figure 9). Care needs to be taken not to breach and cut off the chondral flap, which was previously elevated. An arthroscopic burr (5.5 mm DYONICS ELITE Abrader; Smith & Nephew, Watford, UK) is used to smooth the area of rimplasty (Figure 10). To facilitate healing, care is taken to preserve approximately 1 mm of subchondral bone beneath the cartilage. The chondral flap is carefully mobilized and pressed against the frontal femoral cortex at the resected site (Figure 11).

**FIGURE 12:**
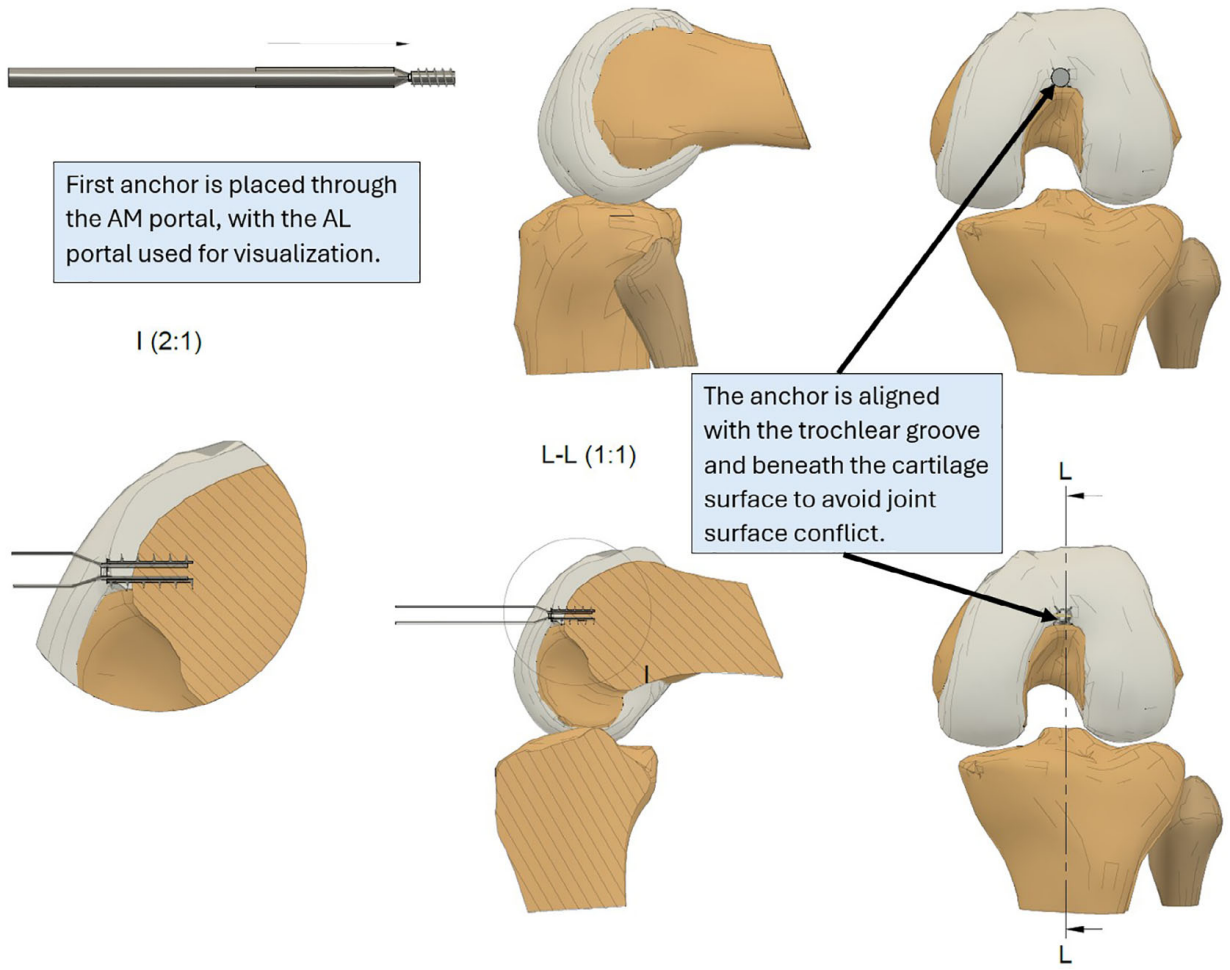
Technical drawings of the left knee in sagittal view (marked with L‐L 1:1). This picture shows the insertion site of the first Healicoil Knotless PK suture anchor (Smith & Nephew, Watford, UK), which will be used to reinsert the previously elevated and mobilized chondral flap to the site of the rimplasty. First anchor placement is performed in “Position I,” with the surgeon facing the patient's head and the operated limb flexed to 90°. The anteromedial portal is used as the working portal and serves for anchor insertion. The anterolateral portal is used as the viewing portal. The area of first anchor placement is further exposed on the drawing on the left side, marked with I (2:1). Arrows point to the location of anchor placement on the transverse view. Drawing on the right down corner shows the exact location of the sagittal slice marked with L‐L (line marked with L). (AL, anterolateral; AM, anteromedial.)

**FIGURE 13:**
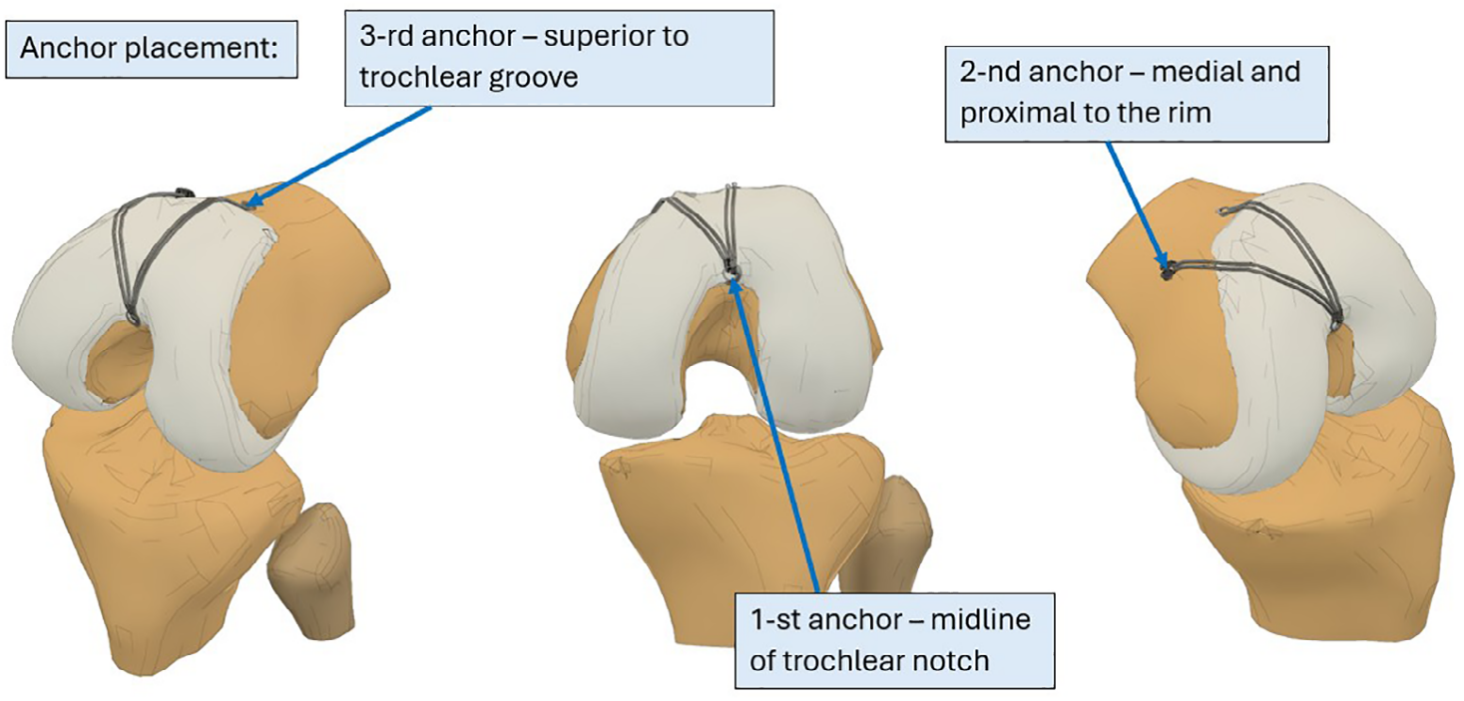
Technical drawings of the knee, showing the placement of all 3 anchors. Two ETHICON Coated VICRYL 6 (Johnson & Johnson International, New Brunswick, NJ) are initially used with the first anchor (4 strands) and then retracted to the suprapatellar pouch. The first anchor is inserted in “Position I,” with the surgeon facing the patient's head and the patient's knee flexed to 90°. In this setting, the anterolateral portal serves as the viewing portal, and the anteromedial portal serves as the working portal. After this step, the surgeon changes position to “Position II,” now facing the patient's feet, with the operated limb in full extension lying on the operating table. In this position, the suprapatellar lateral portal is used as the viewing portal, and the suprapatellar medial portal is used to introduce the second and third Healicoil Knotless PK suture anchors (Smith & Nephew, Watford, UK). The second Healicoil Knotless PK suture anchor (Smith & Nephew, Watford, UK) is placed medially, aligned with the medial femoral condyle. The third anchor is placed lateral to the second anchor, just medial to the trochlear groove. For each anchor, 2 strands of ETHION Coated VICRYL 6 (Johnson & Johnson International, New Brunswick, NJ) are used and tightened to stabilize the chondral flap after rimplasty. The proper placement of anchors is important to achieve stable fixation of the chondral flap.

If the osteochondral flap is rigid, it is mobilized and gently reshaped using an arthroscopic hook or a thin instrument to conform to the trochlear groove. In Position I, the first Healicoil Knotless suture anchor (Smith & Nephew, Watford, UK) is placed through the anteromedial portal, with the AL portal used for visualization. Two ETHICON Coated VICRYL 6 (Johnson & Johnson International, New Brunswick, NJ) sutures are used for this anchor (4 strands are needed for further stabilization: 2 strands for the second and 2 for the third anchor). The anchor is aligned with the trochlear groove and beneath the cartilage surface to avoid joint surface conflict (Figure 12). All sutures are retracted upward to the suprapatellar recess. The surgeon returns to Position II to place the second Healicoil Knotless PK suture anchor (Smith & Nephew, Watford, UK) medially, aligned with the medial femoral condyle. The third anchor is placed lateral to the second anchor, just medial to the trochlear groove. For each anchor, 2 strands of ETHICON Coated VICRYL 6 (retracted from the first anchor) are used. The sutures are tightened to achieve stable fixation of the osteochondral flap (Figure 13). Any irregularities in the subchondral bone are trimmed to ensure a smooth transition between the anterior femoral cortex and the trochlear groove. Joint congruity is assessed by passive flexion and extension and visualized arthroscopically via the AL or lateral suprapatellar portal.

#### Patellar Cartilage Reconstruction

Following this, the PC lesion is explored and treated. The extent of cartilage damage is evaluated using an arthroscopic hook or blunt instrument. Lesion preparation to the subchondral bone is performed with front and sideward chondrectomes (Chondrectom Extended Set, Biovico, Gdynia, Poland) up to the borders of healthy cartilage, and any irregular edges are smoothed with an arthroscopic shaver. Depending on the lesion's location, all portals may serve as working or viewing portals. Next, during dry arthroscopy, the autologous matrix‐induced chondrogenesis technique^22^, ^23^ is applied to the prepared area. Circles (5 × 5 mm) cut from collagen membrane (Chondro‐Gide, Geistlich, Switzerland), presoaked in bone marrow aspirate, are placed on the prepared chondral lesion of the patellar articular surface. The membrane is stabilized with fibrin glue (Tissucol, Baxter, Warsaw, Poland), ensuring no adhesions with the synovium or soft tissue. The joint is reassessed for congruity via passive motion and arthroscopic visualization. After confirming secure fixation and alignment, the incisions are closed without drainage and a layered dressing is applied. Pearls and pitfalls of rimplasty are described in Table 1.

**TABLE 1:** Pearls/Pitfalls of the Presented Technique

Pearls	Pitfalls
1. During rim preparation, the camera and instruments should be positioned in the superior portals. The surgeon should be facing the foot and adjust their position as needed 2. Remove the synovium from the suprapatellar recess to ensure it does not obstruct the visualization during dry arthroscopy 3. When switching surgical portals, guides and cannulas should be used to allow smooth insertion of instruments into the knee joint 4. Appropriate osteotomy direction allows controlled resection of the rim 5. A small burr should be used to smooth the area after rimplasty to ease the transition between the anterior femoral cortex and the medial condyle	1. Improperly positioned surgical portals may hinder free instrument manipulation within the knee joint 2. Care should be taken when introducing the chisel so as not to break or cut through the medial condyle cartilage 3. Sutures should be placed under tension to stabilize the osteochondral flap into the resected area of the rim 4. The chisel should be sharp to cut effortlessly through bone; if the chisel is blunt, it can easily slip and damage the femoral cartilage 5. Rimplasty should be performed first, before AMIC reconstruction of the patellar chondral defect. If AMIC is performed first, visibility can be compromised

AMIC, autologous matrix‐induced chondrogenesis.

## REHABILITATION PROTOCOL

After surgery, the patient starts knee flexion exercises using a continuous passive motion device on the second day, continuing for 4 to 6 hours daily until 2 weeks postoperation. The continuous passive motion should be set to the highest flexion angle tolerable without causing pain. Sutures are removed 2 weeks after surgery. The patient uses elbow crutches with partial weight‐bearing for up to 6 weeks. Return to sports (e.g., running) is permitted only after regaining full range of motion, absence of knee pain or swelling, and when both limbs have comparable strength, endurance, and function.

## DISCUSSION

In individuals without patellar instability, isolated treatment of PC lesions often yields suboptimal outcomes.^24^, ^25^ Rimplasty is intended for patients without clinical signs of patellofemoral instability and with supratrochlear rim height extending 3 mm. In cases of smaller rim deformity, arthroscopic rim resection has been described in the literature.^18^

Trochleoplasty and grooveplasty are techniques for patients with PFJ instability due to pronounced dysplasia, aiming to restore the native anatomy of the femoral trochlea, and are effective in restoring patellofemoral stability.^25^, ^26^, ^27^, ^28^ However, trochleoplasty carries a significant risk of complications.^29^, ^30^ Grooveplasty, a less‐invasive technique, can be a valuable tool in cases of shallow trochlea and patellar instability during initial flexion. However, arthroscopic versions of these techniques, which are technically more demanding, lead to similar outcomes.^31^, ^32^ The technique described by the authors involves a more conservative form of trochleoplasty, limited to remodeling only the entry zone of the femoral trochlear groove to optimize patellofemoral tracking. It is a complex arthroscopic procedure that requires a highly skilled operator and a set of dedicated tools for rim removal and PC reconstruction (Table 2). This arthroscopic procedure addresses the large (>3 mm) supratrochlear rims and isolated patellar chondromalacia. Future treatment strategies might benefit from combining different arthroscopic techniques, surgical navigation and robotic solutions,^33^, ^34^, ^35^, ^36^ and arthroscopic image processing,^37^, ^38^, ^39^ to achieve better biomechanical and clinical outcomes.

**TABLE 2:** Advantages and Disadvantages of the Presented Technique

Advantages	Disadvantages
1. Minimally invasive surgery without the need for a wide arthrotomy of the joint to reconstruct the patellar cartilage 2. Precise preparation of the supratrochlear rim site 3. Accurate assessment of the knee joint, all joint compartments, arthroscopically	1. Technically challenging, requiring high arthroscopic skills 2. Use of dedicated tools for supratrochlear rim removal 3. Changing positions can be demanding for surgeons, requiring them to maintain spatial orientation

## DISCLOSURES

The authors (T.P., B.B., J.B., A.B.) declare that they have no known competing financial interests or personal relationships that could have appeared to influence the work reported in this article.

## FUNDING

Open access publishing facilitated by Ecole polytechnique federale de Lausanne, as part of the Wiley ‐ Ecole polytechnique federale de Lausanne agreement via the Consortium Of Swiss Academic Libraries.
